# Design, Synthesis and Anti-HIV Integrase Evaluation of 4-Oxo-4*H*-quinolizine-3-carboxylic Acid Derivatives

**DOI:** 10.3390/molecules14020868

**Published:** 2009-01-19

**Authors:** Yi-Sheng Xu, Cheng-Chu Zeng, Zi-Guo Jiao, Li-Ming Hu, Ru-gang Zhong

**Affiliations:** 1 College of Life Science and Bio-Engineering, Beijing University of Technology, Beijing 100124, P. R. China; 2 Atmospheric Chemistry & Aerosol Research Department, Chinese Research Academy of Environmental Sciences, Beijing 100012, P. R. China; E-mails: xuys@craes.org.cn (Y-S.X)

**Keywords:** 4-Oxo-4*H*-quinolizine-3-carboxylic acid derivatives, HIV-1 integrase, Mg^2+^ binding, Aryl diketo acids

## Abstract

4-Oxo-4*H*-quinolizine-3-carboxylic acid derivatives bearing sulfamido, carboxylamido, benzimidazole and benzothiazole substituents have been designed and synthesized. The structures of these new compounds were confirmed by ^1^H-NMR, ^13^C- NMR, IR and ESI (or HRMS) spectra. Compounds were screened for possible HIV integrase inhibitory activity.

## Introduction

Human immunodeficiency virus type 1 (HIV-1) is the major pathogen of the infection of acquired immunodeficiency syndrome (AIDS) [1]. In the past two and half decades, various compounds have been developed as medicinal candidates for the treatment of HIV infections aiming at one or several steps of the HIV-1 life cycle [2,3,4,5] such as absorption, entry, fusion, un-coating, reverse transcription, integration, transcription and maturation. Owing to the facts that HIV integrase (IN) is the indispensable enzyme in the replication of the life cycle for the HIV virus and no cellular homologues are found in humans [6,7,8,9], inhibitors targeted selectively at HIV IN are expected to have low cytotoxicity and thus development of HIV IN inhibitors has attracted more and more attention. 

To this end, aryl diketoacids compounds have been reported to be the most promising HIV IN inhibitors [10,11,12]. Structurally, this type of HIV IN inhibitors consists of two main domains, namely, a diketoacid subunit and an aromatic moiety with one or two arylalkyl substituents. The former serves as the pharmaphore and is proposed to sequester the divalent cofactors (Mg^2+^) in the IN active site and thus block access of a host DNA to the integrase [13,14]. Meanwhile, the aromatic moiety as hydrophobic domain, typically an indole or benzene moiety, is responsible for the selectivity to the strand transfer (ST) reaction [15,16]. 

**Figure 1:**
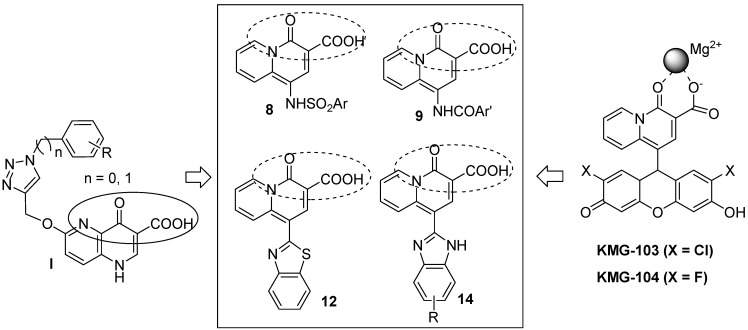
The design of 4-oxo-quinolizine-3-carboxylic acid derivatives as potential HIV IN inhibitors.

As part of our continuing efforts directed towards the development of potential HIV-1 integrase inhibitors, we have investigated the chemical and electrochemical synthesis of styrylquinolines [17], quinoxalones [18,19] and polyhydroxylated aromatics [20,21]. Based on the structural characteristics of diketo acid-type compounds and their molecular mechanism as HIV IN inhibitors, very recently, a series of 4-oxo-1,4-dihydro-1,5-naphthyridine-3-carboxylic acids have been designed and synthesized by our group (see I, Figure 1) [22]. In view of the fact that the 4-oxo-quinolizine-3-carboxylic acid moiety displays high selectivity and binding ability to Mg(II) (KMG-103 and KMG-104, Figure 1) [23,24] and other derivatives show antibacterial and antimicrobial activities [25,26,27], in the present work, we further modified the main core via addition of a bulky group, such as an aryl sulfonamido, aryl carboxylamido, benzimidazole or benzothiazole chosen as the side hydrophobic chain and placed at the C-1 position of the 4-oxo-quinolizine-3-carboxylic acid scaffold. Thus, a series of 4-oxo-quinolizine-3-carboxylic acid derivatives **8, 9, 12 **and **14** were synthesized. Results show that, although these compounds could bind Mg^2+^ in CH_3_CN solution, no obvious anti-HIV IN activity is observed.

## Results and Discussion

### Synthesis of 4-oxo-4H-quinolizine-3-carboxylic acid derivatives **8, 9, 12 **and **14**

**Scheme 1:**
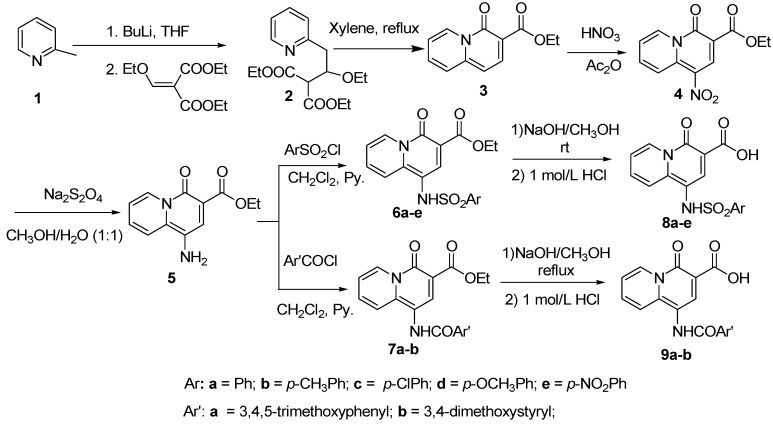
The synthesis of 4-oxo-4H-quinolizine-3-carboxylic acid derivatives **8** and **9**.

The synthesis of 4-oxo-4*H*-quinolizine-3-carboxylic acid derivatives **8** and **9** is described in Scheme 1. Ethyl 4-oxo-4*H*-quinolizine-3-carboxylate (**3**) was synthesized in good yield starting from 2-methyl-pyridine according to a literature procedure [24]. Nitration of **3** afforded compound **4**, which was reduced by Na_2_S_2_O_4_ to generate the key intermediate ethyl 1-amino-4-oxo-4*H*-quinolizine-3-carboxylate (**5**). In the presence of pyridine, compound **5** was treated with sulfonyl chloride or acyl chloride to give compounds **6** and **7**, respectively. The target 4-oxo-4*H*-quinolizine-3-carboxylic acids **8** and **9** were finally obtained after hydrolysis in NaOH/methanol followed by neutralization. During the nitration procedure, the speed of addition of nitric acid should be carefully controlled, otherwise, the reaction mixture would become red and the amount of by-products increased. In addition, Na_2_S_2_O_4_ was used to reduce nitro group and generated key intermediate **5**, due to the benign reaction conditions (usually at room temperature using water as solvent) and simple workup. 

**Scheme 2:**
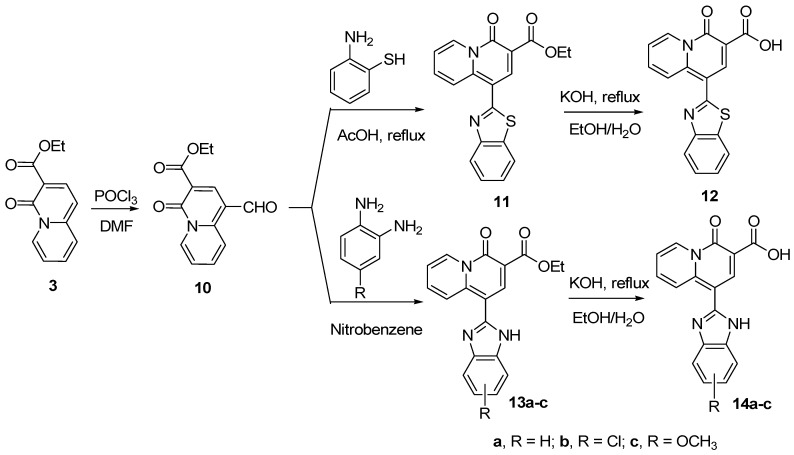
Synthesis of 4-oxo-4*H*-quinolizine-3-carboxylic acid derivatives **12** and **14**.

With **3** in hand, we then synthesized benzothiazole- and benzimidazole-substituted 4-oxo-4*H*-quinolizine-3-carboxylic acid derivatives **12** and **14**. As shown in Scheme 2, reaction of compound **3** with DMF and POCl_3_ generated intermediate **10** [24], which reacted with 2-aminothiophenol [28,29] followed by subsequent hydrolysis to generate 1-(benzothiazole-2-yl)-4-oxo-4*H*-quinolizine-3-carboxylic acid (**12**). Similarly, compound **10** underwent oxidative condensation with *o*-phenylene-diamine derivatives in nitrobenzene [30] yielded compounds **13a-c**, which were hydrolyzed [31] to obtain 1-(benzimidazole-2-yl)-4-oxo-4*H*-quinolizine-3-carboxylic acids **14a-c**. It should be pointed out that two benzimidazole isomers were formed for each of **13b-c **and **14b-c**, that could not be separated each other through conventional procedures. 

It is noteworthy that the conditions for the hydrolysis of **11** and **13 **were not similar to those used for the synthesis of **8** and **9.** The reaction proceeded very slowly in the NaOH/MeOH system. For example, after 48 hours stirring at room temperature, the reaction mixtures were still slurries and most of the starting materials were present in the reaction system. Consequently, KOH in 80% aqueous ethanol was used for the synthesis of **12** and **14. **As the reaction proceeded the slurry of the reaction mixture turned clear and the products precipitated when 1 mol·L^-1^aqueous HCl was added. 

### Structural analysis

The structures of all new 4-oxo-4*H*-quinolizine-3-carboxylic acid derivatives **6-14** were confirmed by ^1^H-NMR, ^13^C-NMR, IR and ESI-MS (or HRMS) spectra. In the ^1^H-NMR spectra of ethyl 4-oxo-4*H*-quinolizine-3-carboxylates **6**, **7**, **11**, **13**, the 4-oxo-4*H*-quinolizine ring proton signals appeared at lower field compared with those of the benzene ring. For example, in the ^1^H-NMR spectra of **6b**, the two doublets at 9.43 ppm and 8.28 ppm were ascribed to the protons at C-5 and C-8 of the 4-oxo-4*H*-quinolizine ring, respectively, while the protons at C-6 and C-7 exhibited two double doublets at 7.76 ppm and 7.29 ppm. The proton at C-2 resided at 7.63 ppm. A high field AB system (7.31 ppm and 7.64 ppm) corresponded to the signals of the four benzene ring protons.

In the ^1^H-NMR of target compounds **8**, **9**, **12**, **14a**, one sharp singlet in the δ 13.7-14.1 range was observed, which disappeared after the D_2_O exchange and was consequently attributed to the COOH proton. For example, the proton signals of the COOH groups of compounds **8a** and **14a** were located at 13.7 ppm and 13.8 ppm, respectively. In addition, compound **14a** has a strong singlet at δ 13.7 ppm, which demonstrated the existence of a N-H proton in the benzimidazole ring. In the ^13^C-NMR spectra of these compounds one C=O peak at about 166 ppm and one around 159 ppm corresponded to the signals of the 3-carboxylic acid and carbonyl groups in the quinolizine rings. The IR spectra of these compounds further support their postulated structures. There were several medium broad bands at 3,200~3,400 cm^-1^ corresponding to O-H and N-H bond and strong bands at 1,690~1,730 cm^-1^ due to C=O bonds. The mass spectra of compounds **8c**, **13b** and **14b** exhibited a M+2 signal, whose intensity was 1/3 of the molecular ion signal, and corresponding to the Cl isotopes in these compounds.

### Preliminary investigation of Mg^2+^ binding

**Figure 2:**
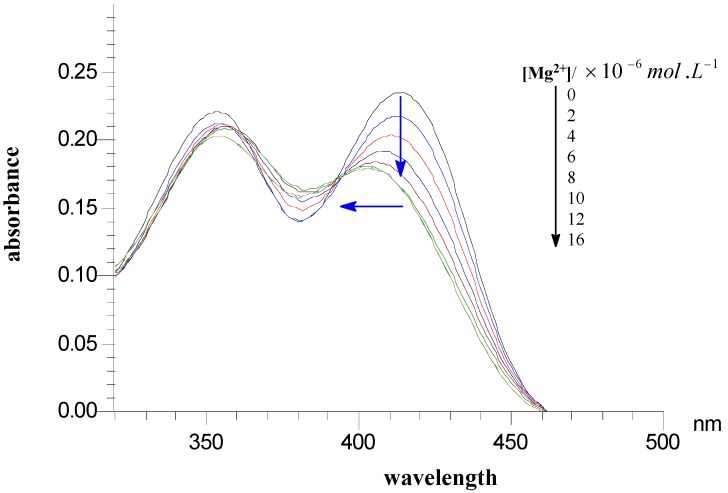
Changes of UV-vis spectra with **14a** in CH_3_CN on addition of 0-16×10^-6^ mol·L^-1^ of [Mg^2+^] at 25 °C.

**Figure 3:**
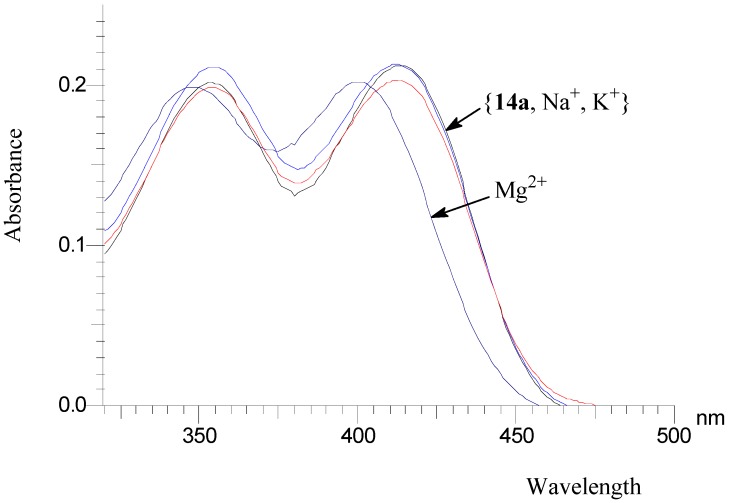
UV-Vis absorption spectra of free **14a** and **14a** in the presence of K^+^, Na^+^ or Mg^2+^ ion in CH_3_CN solution.

To elucidate a two-metal inhibition model, very recently, Kawasuji *et al* [32] performed metal titration studies of 2-hydroxy-3-heteroarylacrylic acid derivatives and observed a two-step shift of the UV-Vis spectra upon the addition of Mg^2+^ if these compounds exhibited HIV-IN inhibitory activity. On the contrary, simple change of UV-Vis absorption curves or no shift was found for their mutated compounds which do not inhibit HIV integrase. Inspirited by this assumption, preliminary ion binding experiments were also carried out by UV-Vis spectroscopy, using compound **14a** as an example. Due to the low solubility of **14a** in water, CH_3_CN was used to prepare the stock solution for the UV-Vis measurements. As shown in Figure 2, for the free compound **14a**, two intense absorption bands at 352 nm and 414 nm were observed in the 320 – 550 nm range. As the Mg^2+^ concentration (as perchlorate salt) increased, the absorption intensity of the peak at 414 nm first decreased and then the wavelength shifted blue to around 402 nm. Figure 3 further demonstrated the selectivity of compound **14a** to Mg^2+^. In the presence of a 100-fold equivalent amount of Na^+^ or K^+^, no obvious change was observed. However, the Mg^2+^ induced a blue shift of the absorption curve of compound **14a**. Similar behaviors were also observed for other divalent metals, including Mn^2+^, Co^2+^, Cu^2+^, Ca^2+^ and Ni^2+^ (not shown in Figure 3).

Conditions: [Mg^2+^] =2 × 10^-3^ M. [**14a**] = 2 × 10^-5^ MBlack: 0×10^-6^ mol·L^-1^, blue: 2×10^-6^ mol·L^-1^, red: 4×10^-6^ mol·L^-1^, navy: 6×10^-6^ mol·L^-1^, brown: 8×10^-6^ mol·L^-1^, green: 10×10^-6^ mol·L^-1^, dark cyan: 12×10^-6^ mol·L^-1^, brass: 16×10^-6^ mol.L^-1^.

The titration experiment of **14a** with Mg^2+^ further demonstrated that the binding ratio is 1:1, which is consistent with that of KMG-103 and KMG-104 to Mg^2+^, indicating that the introduction of benzimidazole substituent on the C-1 position of the 4-oxo-4*H*-quinolizine-3-carboxylic acid scaffold does not affect its Mg^2+ ^binding ability. In a word, a two-step shift of the absorption curve of compound **14a** upon the addition of Mg^2+^ was observed, similar to 2-hydroxy-3-heteroaryl acrylic acid derivatives reported by Kawasuji *et al* [32]. These UV-Vis results indicated that our compounds might interact with metal ions (Mg^2+^) in the active site of HIV IN and thus offer a piece of *in vitro* indication of possible HIV IN inhibitory activity of these new compounds, in spite of no strict demonstration of correction between the inhibitory activity and the metal affinity.

Conditions: [**14a**] = 2 × 10^-5^ M; [Mg^2+^] = [K^+^] = [Na^+^] = 2×10^-3^ M; Black: **14a**, blue: Na^+^ complex, red: K^+^ complex, navy: Mg^2+^ complex.

### HIV integrase inhibitory activities of 4-oxo-4H-quinolizine-3-carboxylic acid derivatives

The target compounds **8**, **9**, **12**, **14** were screened against purified HIV IN to determine any potential inhibitory activity. Thus, a synthesized 30 oligonucleotide and a 20 oligonucleotide were used as donor DNA and target DNA, respectively, which were purchased from Shanghai Sangon Biological Engineering Technology & Services Co. Ltd. The donor DNA was biotinylated at the 5′ end (5′ bio-DNA) and immobilized on streptavidin-coated 96-well microtiter plates. To the donor DNA streptavidin-coated 96-well microtiter plate were added test sample, 3' digoxygenin-labeled target DNA (3' dig-DNA) and recombinant HIV integrase and subjected to incubation in optimized condition.

The plate was further washed with phosphate-buffered saline (PBS) containing 0.1% Tween^®^-20 and anti-digoxigenin-peroxidase (POD, 100 µL) was added to each well. After incubation at 37°C for 60 min, the plate was washed with PBS containing 0.1% Tween^®^-20. The digoxygenin-labeled products were visualized by adding a POD substrate, tetramethylbenzidine (TMB). The colorimetric reaction was stopped by addition of 0.5 mol/L H_2_SO_4 _(100 µL), and the absorbance was measured at 450 nm on a microplate reader. The total inhibitory effect (including inhibiting 3′ process reaction and strand transfer reaction) were obtained by comparing the absorbance of drug group with control group (without test compound). As shown in Table 1, the values of IC_50_ of these compounds are more than 100 μg/mL, which mean a low inhibitory activity to HIV integrase.

The low HIV IN inhibitory activity of these designed 4-oxo-4*H*-quinolizine-3-carboxylic acid derivatives might stem from various cofactors. Structurally, compounds **8**, **9**, **12 **and **14 **possess a chelating scaffold suitable for binding only one ion. However, a two-metal chelating scaffold to form respectively a five-membered and a six-membered system is required according to the two-metal coordinating model for diketoacid-type HIV IN inhibitors. Therefore, the selective binding to Mg^2+^ is not enough, the two-metal binding scaffold is also necessary in the design of diketoacid-type HIV IN inhibitors. In addition, an appropriate substituent at appropriate position of the chelating scaffold is also essential to ensure potential inhibitory activity. 

**Table 1:** Integrase inhibitory activity data of 4-oxo-4*H*-quinolizine-3-carboxylic acid derivatives.

Sample	Initial concentration (μg/mL)	IC_50_ (μg/mL)	Sample	Initial concentration (μg/mL)	IC_50_ (μg/mL)
S-y^1^	12	0.56	**9a**	100	-^2^
**8a**	100	-^2^	**9b**	100	-^2^
**8b**	100	-^2^	**12**	100	-^2^
**8c**	100	-^2^	**14a**	100	-^2^
**8d**	100	-^2^	**14b**	100	-^2^
**8e**	100	-^2^	**14c**	100	-^2^

^1^ S-y provided by the Shanghai Institute of Organic Chemistry was used as positive contrast. ^2^ “-” indicates that the HIV-IN inhibitory effect was less than 50% at the initial concentration.

## Conclusions

Aryl diketoacid-containing compounds were demonstrated to be the most promising HIV IN inhibitors. Based on the molecular mechanism of aryl diketoacids as HIV IN inhibitors and the selective binding to Mg^2+^ of 4-oxo-4*H*-quinolizine-3-carboxylic acid unit, a series of 4-oxo-4*H*-quinolizine-3-carboxylic acid derivatives, substituted by sulfonoamido, carboxylamido, benzimidazole and benzothiazole groups, were designed and synthesized as diketoacid bioisosters. Their anti-HIV IN activity was evaluated and no obvious inhibitory activities were observed. Based on the present results, in the course of our coming works on development of HIV IN inhibitors, we would have a concept that new types of HIV IN inhibitors would incorporate a two-metal chelating scaffold and a appreciate hydrophobic domain. Moreover, the two-metal chelating scaffold could have an obvious binding affinity to Mg^2+^. 

## Experimental

### General

All melting points (m.p.) were measured with a XT4A electrothermal apparatus equipped with a microscope and are uncorrected. Infrared spectra (IR) were recorded as thin films on KBr plates with a Bruker IR spectrophotometer and are expressed in *v* (cm^-1^). ^1^H- and ^13^C-NMR spectra were recorded with an AV 400 M Bruker spectrometer (400 MHz ^1^H frequency, 100 MHz ^13^C frequency). Chemical shifts were measured in CDCl_3_ or DMSO-*d_6_* with TMS as internal reference. The MS spectra (ESI) were recorded on a Bruker Esquire 6000 mass spectrometer. HRMS spectra were recorded in the negative ion mode using APEX II FT-ICRMS of Bruker Daltonics Inc. All solvents were of commercial quality and were dried and purified by standard procedures. Compounds **2**, **3, 4, 10** were synthesized according to the literature procedure [24].

### General procedure for the synthesis of ethyl 1-(substituted phenylsulfonamido)-4-oxo-4H-quinolizine-3-carboxylates **6**

Compound **4** (1.97 g, 7.5 mmol) was suspended in a 1:1 mixture of methanol and water (100 mL) in a 250 mL flask. Na_2_S_2_O_4_ (7.8 g，45 mmol) was then added in one portion at room temperature and the mixture was stirred for about 2 hours. After the reaction, the organic compound was extracted three times using CH_2_Cl_2_ and dried over anhydrous Na_2_SO_4_. After filtration of the Na_2_SO_4_, the solvent was removed under reduced pressure to give 1.0 g (57%) of **5** as a red solid, which was used for the following reactions without further purification. The appropriate substituted sulfonyl chloride (3.44 mmol) in CH_2_Cl_2_ (30 mL) was added dropwise to a CH_2_Cl_2_ solution (30 mL) containing **5** (3.44 mmol) and dry pyridine (0.3 mL). The solution changed from red to dark and TLC showed the formation of new compounds with red fluorescence emission. After the reaction, the solution was washed with water and the organic layer was dried over anhydrous MgSO_4_. After filtration of the MgSO_4_, the solvent was removed under reduced pressure and the residue was recrystallized by ethyl acetate to give the desired products **6** as yellow solids.

*Ethyl 1-phenylsulfonamido-4-oxo-4H-quinolizine-3-carboxylate* (**6a**): Yield 72%. m.p.: 207-209 °C; ^1^H-NMR (CDCl_3_): *δ* 1.34 (t, 3H, *J* = 7.1 Hz, CH_3_), 4.32 (q, 2H, *J* = 7.1 Hz, OCH_2_), 7.25 (dd, 1H, *J* = 7.7 Hz, *J* = 5.9 Hz), 7.48~7.91 (m, 8H, Ar-H), 9.42 (d, 1H, *J* = 7.1 Hz, ); ^13^C-NMR (CDCl_3_): *δ* 14.4, 60.9, 105.6, 106.9, 117.3, 122.0, 128.9, 129.3, 130.1, 134.6, 135.5, 138.4, 143.9, 147.2, 154.7, 164.5; IR: *ν* 3491, 3139, 2981, 1734, 1698, 1493 cm^-1^; MS (ESI): *m/z* 370.9 [M - H]^-^.

*Ethyl 1-p-methylphenylsulfonamido-4-oxo-4H-quinolizine-3-carboxylate* (**6b**): Yield 60%. m.p.: 115-117 °C; ^1^H-NMR (CDCl_3_): *δ* 1.31 (t, 3H, *J* = 7.2 Hz, CH_3_), 2.45 (s, 3H, PhCH_3_), 4.30 (q, 2H, *J* = 7.2 Hz, CH_2_), 6.38 (s, 1H, NH), 7.29~7.33 (m, 3H, Ar-H), 7.64(s, 1H, Ar-H), 7.65(d, 2H, *J* = 7.6 Hz, Ar), 7.77 (dd, 1H, *J* = 7.5 Hz, J = 8.0 Hz, Ar-H), 8.28 (d, 1H, *J* = 8.8 Hz, Ar-H), 9.43 (d, 1H, *J* = 7.1 Hz); ^13^C-NMR (CDCl_3_): *δ* 14.6, 21.8, 61.1, 104.9, 108.3, 117.7, 122.9, 128.0, 130.1, 130.1, 135.4, 135.5, 141.5, 144.7, 146.9, 155.1, 165.3; IR: *ν* 3452, 3164, 2972, 1718, 1629, 1490 cm^-1^; MS (ESI): *m/z* 387.0 [M + H]^+^.

*Ethyl 1-p-chloro-phenylsulfonamido-4-oxo-4H-quinolizine-3-carboxylate* (**6c**): Yield 73%. m.p.: 210-212°C; ^1^H-NMR (DMSO-*d*_6_): *δ* 1.24 (t, 3H, *J* = 7.6 Hz, CH_3_), 4.18 (q, 2H, *J* = 7.6 Hz, CH_2_), 7.54 (s, 1H, Ar-H), 7.55 (dd, 1H, *J* = 7.2 Hz, *J* = 6.4 Hz, Ar-H), 7.68 (d, 2H, *J* = 8.8 Hz, Ar-H), 7.73 (d, 2H, *J* = 8.8 Hz, Ar-H), 7.97-8.05 (m, 2H, Ar-H), 9.29 (d, 1H, *J* = 7.2 Hz, Ar-H); ^13^C-NMR (DMSO-*d*_6_): *δ* 14.6, 60.3, 103.1, 108.5, 118.7, 122.4, 129.7, 129.9, 130.0, 136.6, 138.0, 138.6, 140.1, 146.0, 154.0, 164.5; IR: *ν* 3445, 3155, 2972, 1715, 1494 cm^-1^; MS (ESI): *m/z* 404.9 [M -1]^-^, 428.8 [M + Na]^+^.

*Ethyl 1-p-methyloxyphenylsulfonamido-4-oxo-4H-quinolizine-3-carboxylate* (**6d**): Yield 92%. m.p.: 236-238°C; ^1^H NMR (DMSO-*d*_6_): *δ* 1.19 (t, 3H, *J* = 6.9 Hz, CH_3_), 3.82 (s, 3H, OCH_3_), 4.11 (q, 2H, *J* = 7.0, CH_2_), 7.06 (d, 2H, *J* = 8.6, Ar-H), 7.49-7.51 (m, 2H, Ar-H), 7.60 (d, 2H, *J* = 8.6 Hz, Ar-H), 7.90-8.01 (m, 2H, Ar-H), 9.24 (d, 1H, *J* = 7.2 Hz, Ar-H), 9.58 (s, 1H, NH); ^13^C NMR (DMSO-*d*_6_): *δ* 14.5, 56.1, 60.4, 103.2, 109.1, 114.9, 118.7, 122.5, 129.8, 129.9, 130.6, 136.3, 140.3, 146.0, 154.1, 163.2, 164.6; IR: *ν* 3442, 3170, 2927, 1716, 1650, 1494 cm^-1^; MS (ESI): *m/z* 424.9 [M+Na]^+^.

*Ethyl 1-p-nitrophenylsulfonamido-4-oxo-4H-quinolizine-3-carboxylate* (**6e**): Yield: 76%. m.p.: 265-267°C; ^1^H-NMR (DMSO-*d*_6_): *δ* 1.15 (t, 3H, *J* = 7.2 Hz, CH_3_), 4.1 (q, 2H, *J* = 7.2 Hz, CH_2_), 7.51 (s, 1H, Ar-H), 7.49-7.53 (m, 1H, Ar-H), 7.94-7.98 (m, 4H, Ar-H), 8.39 (d, 2H, *J* = 8.8 Hz, Ar-H), 9.27 (d, 1H, *J* = 7.2 Hz, Ar-H), 10.20 (s, 1H, NH); ^13^C-NMR (DMSO-*d*_6_): *δ* 14.5, 60.4, 103.1, 108.0, 118.8, 122.2, 125.0, 129.4, 130.1, 136.8, 140.1, 144.8, 145.8, 150.4, 154.1, 164.6; IR: *ν* 3446, 3204, 2981, 1727, 1627, 1497, 1233 cm^-1^; MS (ESI): *m/z* 439.9 [M + Na]^+^.

### General procedure for the synthesis of 1-(substituted phenylsulfonamido)-4-oxo-4H-quinolizine-3-carboxylic acids **8**

To a suspension of **6** (1.2 mmol) in methanol (15 mL) was added 3 mol/L of NaOH (0.9 mL). The reaction mixture was stirred overnight, followed by evaporation of the solvent under reduced pressure. The remaining solid was dissolved in water (20 mL) and the clear solution was acidified with 1 mol/L HCl to pH 1. The resulting suspension was filtered and dried under vacuum over P_2_O_5_ to give yellowish solids.

*1-Phenylsulfonamido-4-oxo-4H-quinolizine-3-carboxylic acid* (**8a**): Yield 82%. m.p.: 245-247°C; ^1^H- NMR (DMSO-*d*_6_): *δ* 7.51-7.55 (m, 2H, Ar-H), 7.63-7.66 (m, 5H, Ar-H), 8.00-8.09 (m, 2H, Ar-H), 9.29 (d, 1H, *J* = 7.2 Hz, Ar-H), 10.05 (s, 1H, NH), 13.71 (s, 1H, COOH); ^13^C-NMR (DMSO-*d*_6_): *δ* 103.7, 113.0, 120.2, 122.9, 127.6, 129.8, 133.8, 136.9, 138.6, 138.9, 145.0, 158.9, 165.6; IR: *ν* 3441, 3293, 3116, 1727, 1616, 1495, 1446, 1365, 1327, 1159 cm^-1^; MS (ESI): *m/z* 344.9 [M + H]^+^, 366.9 [M + Na]^+^.

*1-p-Methylphenylsulfonamido-4-oxo-4H-quinolizine-3-carboxylic acid* (**8b**): Yield 75%. m.p.: > 300 °C; ^1^H-NMR (DMSO-*d*_6_): *δ* 2.35 (s, 3H, CH_3_), 7.33 (d, 2H, *J* = 8.1 Hz, Ar-H), 7.54 (d, 2H, *J* = 8.1 Hz, Ar-H), 7.64 (dd, 1H, *J* = 7.0 Hz, Ar-H), 7.67 (s, 1H, Ar-H), 8.03 (dd, 1H, *J* = 7.2 Hz, *J* = 8.4 Hz, Ar-H), 8.11 (d, 1H, *J* = 8.8 Hz, Ar-H), 9.29 (d, 1H, *J* = 7.1 Hz, Ar-H);^ 13^C-NMR (DMSO-*d*_6_): *δ* 21.5, 103.7, 113.3, 120.2, 123.0, 127.6, 129.8, 130.2, 136.2, 136.8, 138.5, 144.1, 145.0, 158.9, 165.7; IR: *ν* 3265, 3071, 1725, 1619, 1492, 1448, 1292, 1160, 816 cm^-1^; HRMS: *m/z* calcd for C_17_H_13_ N_2_O_5_S: 357.0551; found: 357.0550.

*1-p-Chlorophenylsulfonamido-4-oxo-4H-quinolizine-3-carboxylic acid* (**8c**): Yield 79%. mp > 300°C; ^1^H-NMR (DMSO-*d*_6_): *δ* 7.65-7.72 (m, 6H, Ar-H), 8.09-8.15 (m, 2H, Ar-H), 9.36 (d, 1H, *J* = 7.1 Hz, Ar-H), 10.22 (s, 1H, NH), 13.70 (s, 1H, OH); ^13^C-NMR (DMSO-*d*_6_): *δ* 103.7, 112.7, 120.3, 122.9, 129.6, 129.9, 129.9, 137.0, 137.8, 138.5, 138.6, 145.0, 158.9, 165.7; IR: *ν* 3446, 3265, 2924, 1720, 1489, 1163 cm^-1^; MS (ESI): *m/z* 376.9 [M-H]^-^.

*1-p-Methoxyphenylsulfonamido-4-oxo-4H-quinolizine-3-carboxylic acid* (**8d**): Yield 86%. mp > 300°C; ^1^H-NMR (DMSO-*d*_6_): *δ* 3.79 (s, 3H, CH_3_), 7.03 (d, 2H, *J* = 8.8 Hz, Ar-H), 7.57 (d, 2H, *J* = 8.8 Hz, Ar-H), 7.65 (dd, 1H, *J* = 10.0 Hz, *J* = 7.2 Hz, Ar-H), 7.68 (s, 1H, Ar-H), 8.03 (dd, 1H, *J* = 8.6 Hz, *J* = 7.0, Ar-H), 8.10 (d, 1H, *J* = 8.8Hz, Ar-H), 9.29 (d, 1H, *J* = 7.2Hz, Ar-H), 9.86 (s, 1H, NH); ^13^C- NMR (DMSO-*d*_6_): *δ* 56.2, 103.7, 113.3, 114.9, 120.2, 123.0, 129.7, 129.8, 130.5, 136.8, 138.6, 145.1, 158.9, 163.3, 165.7; IR: *ν* 3248, 2918, 2849, 1724, 1618, 1597, 1496, 1450, 1294, 1157, 836 cm^-1^; MS (ESI): *m/z* 375.0 [M+H]^+^.

*1-p-Nitrophenylsulfonamido-4-oxo-4H-quinolizine-3-carboxylic acid* (**8e**): Yield 82%; mp > 300°C; ^1^H-NMR (DMSO-*d*_6_): *δ* 7.60-7.80 (m, 1H, Ar-H), 7.73 (s, 1H, Ar-H), 7.94 (d, 2H, *J* = 8.0 Hz, Ar-H), 8.02-8.05 (m, 2H, Ar-H), 8.35 (d, 2H, *J* = 8.0 Hz, Ar-H), 9.32 (d, 1H, *J* = 6.4 Hz, Ar-H), 10.49 (s, 1H, NH), 13.68 (s, 1H, OH); ^13^C-NMR (DMSO-*d*_6_): *δ* 103.8, 112.3, 120.3, 122.6, 125.1, 129.3, 129.9, 137.2, 138.6, 144.7, 144.8, 150.4, 158.9, 165.7; IR: *ν* 3454, 3234, 3082, 1715, 1488, 1167, 786 cm^-1^; MS (ESI): *m/z* 387.8 [M-H]^-^.

### General procedure for the synthesis of **7a,b**

To a solution of 3,4,5-trimethoxylbenzoic acid or 3,4-dimethoxycinnamic acid (4 mmol) in ether (20 mL) was added PCl_5 _(20 mL) in one portion. The reaction mixture was heated to reflux for about 4 hours and then cooled to room temperature. After maintaining in a refrigerator for about 1 hour, a white precipitate formed which was filtered and recrystallized from cold ether to give the corresponding carboxylic chloride. The newly-synthesized acyl chloride dissolved in CH_2_Cl_2_ (20 mL) was added dropwise to a mixture of compound **5** (3 mmol) and triethylamine (3 mmol) in CH_2_Cl_2_ (20 mL). The reaction solution was stirred overnight at room temperature. After the reaction, the solution was washed with water and the organic layer was dried over anhydrous MgSO_4_. After filtration of the MgSO_4_, the solvent was removed under reduced pressure and the residue was purified by recrystallization from ethyl acetate to give **7a** or **7b** as a yellow solid.

*Ethyl 1-(3,4,5-trimethoxyphenylcarbonylamino)-4-oxo-4H-quinolizine-3-carboxylate* (**7a**): Yield 40%; mp 93°C; ^1^H-NMR (CDCl_3_): *δ* 1.29 (t, 3H, *J* = 7.2 Hz, CH_3_), 3.92 (s, 3H, OCH_3_), 3.95 (s, 6H, OCH_3_), 4.26 (q, 2H, *J* = 7.2 Hz, OCH_2_), 7.11 (dd, 1H, *J* = 6.8 Hz, *J* = 7.2 Hz, Ar-H), 7.39 (s, 2H, Ph-H), 7.57 (dd, 1H, *J* = 8.8 Hz, *J* = 6.8 Hz, Ar-H), 7.66 (d, 1H, *J* = 8.8 Hz, Ar-H), 8.08 (s, 1H, Ar-H), 8.66 (bs, 1H, NH), 9.17 (d, 1H, *J* = 7.2 Hz, Ar-H);^ 13^C-NMR (CDCl_3_): δ = 14.4, 56.4, 60.9, 61.0, 105.1, 105.1, 110.6, 116.9, 122.0, 128.4, 129.4, 134.2, 140.2, 141.5, 143.7, 153.3, 154.9, 165.3, 166.9; IR: *ν* 3462, 3328, 2939, 1720, 1678, 1587, 1487, 1336, 1216, 1126, 1009, 772 cm^-1^; MS (ESI): *m/z* 449.0 [M + Na]^+^.

*Ethyl 1-[3-(3,4-dimethoxyphenyl)acrylamido]-4-oxo-4H-quinolizine-3-carboxylate* (**7b**): Yield 85%; m.p.: 105-107°C; ^1^H-NMR (DMSO-*d*_6_): *δ* 1.30 (t, 3H, *J* = 7.2 Hz, CH_3_), 3.81 (s, 3H, OCH_3_), 3.83 (s, 3H, OCH_3_), 4.26 (q, 2H, J = 7.2 Hz, CH_2_), 6.81 (d, 1H, *J* = 15.6 Hz, CH=CH), 7.03 (d, 1H, *J* = 8.4 Hz, Ar-H), 7.21 (d, 1H, *J* = 8.4 Hz, Ar-H), 7.26 (s, 1H, Ar-H), 7.51 (dd, 1H, *J* = 6.4 Hz, *J* = 7.2 Hz, Ar-H), 7.55 (d, 1H, *J* = 15.6 Hz, Ar-H), 7.86 (d, 1H, J = 8.8 Hz, Ar-H), 7.96 (dd, 1H, *J* = 7.2 Hz, *J* = 8.0 Hz, Ar-H), 8.26 (s, 1H, Ar-H), 9.30 (d, 1H, *J* = 7.2 Hz, Ar-H), 9.89 (s, 1H, NH); ^13^C-NMR (DMSO-*d*_6_): *δ* 14.8, 55.9, 56.0, 60.5, 103.7, 110.6, 111.1, 112.2, 118.2, 119.4, 122.3, 122.4, 127.9, 129.8, 135.8, 139.0, 141.1, 143.1, 149.4, 150.9, 154.1, 165.3, 165.9; IR: *ν* 3436, 3226, 2935, 1702, 1677, 1595, 1491, 1377, 1236, 1114, 1026, 768 cm^-1^; MS (ESI): *m/z* 445.0 [M + Na]^+^.

### General procedure for the synthesis of **9a,b**

To a suspension of **7a** or **7b** (0.5 mmol) in 80% methanol (20 mL) was added NaOH (5 mmol). The reaction mixture was heated to reflux till the starting **7a** or **7b** disappeared according to monitoring by TLC. After evaporation of the solvent under reduced pressure, the remaining solid was dissolved in water (20 mL) and the clear solution was acidified with 1 mol/L HCl to pH 1 and the resulting suspension extracted with CH_2_Cl_2_. The combined organic layer was dried over anhydrous MgSO_4_ followed by filtration. The desired **9a** or **9b** were finally obtained as solids by column chromatography (eluents: CH_3_OH/CHCl_3_ = 1:30)

*1-(3,4,5-Trimethoxyphenylcarbonylamino)-4-oxo-4H-quinolizine-3-carboxylic acid* (**9a**): Yield 92%; m.p.: 280-282°C; ^1^H-NMR (DMSO-*d*_6_): *δ* 3.67 (s, 3H, OCH_3_), 3.80 (s, 6H, OCH_3_), 7.33 (s, 2H, Ar-H), 7.64 (dd, 1H, *J* = 7.2 Hz, *J* = 6.8 Hz, Ar-H), 7.94 (d, 1H, *J* = 8.8 Hz, Ar-H), 8.02 (dd, 1H, *J* = 6.8 Hz, *J* =8.8 Hz, Ar-H ), 8.29 (s, 1H, Ar-H), 9.30 (d, 1H, *J* = 7.2 Hz, Ar-H), 10.29 (s, 1H, NH), 13.96 (s, 1H, COOH);^ 13^C-NMR (DMSO-*d*_6_): *δ* 56.6, 60.6, 103.9, 105.9, 115.2, 105.9, 115.2, 120.2, 123.3, 129.1, 129.7, 136.6, 138.2, 141.0, 143.0, 153.2, 159.0, 166.1, 166.5; IR: *ν* 3440, 3115, 2924, 2853, 1696, 1657, 1615, 1587, 1496, 1453, 1364, 1292, 1123 cm^-1^; MS (ESI): *m/z* 397.0 [M - H]^-^.

*1-(3,4-Dimethoxystyrylcarboxylamino)-4-oxo-4H-quinolizine-3-carboxylic acid* (**9b**): Yield 91%; m.p.: 306-308°C; ^1^H-NMR (DMSO-*d*_6_): *δ* 3.82 (s, 3H, OCH_3_), 3.84 (s, 3H, OCH_3_), 6.84 (d, 1H, *J* = 16.0 Hz, CH=CH), 7.03 (d, 1H, *J* = 8.4 Hz, Ar-H), 7.23 (d, 1H, *J* = 8.4 Hz, Ar-H), 7.27 (s, 1H, Ar-H), 7.58 (d, 1H, *J* = 16.0 Hz, Ar-H), 7.70-7.74 (m, 1H, Ar-H), 8.10-8.12 (m, 2H, Ar-H), 8.44 (s, 1H, Ar-H), 9.37 (d, 1H, *J* = 7.2 Hz, Ar-H), 10.11 (s, 1H, NH), 14.13 (s, 1H, OH); ^13^C-NMR (DMSO-*d*_6_): *δ* 56.0, 56.1, 103.9, 110.9, 112.4, 115.5, 119.3, 120.0, 122.3, 122.8, 127.9, 136.3, 136.9, 141.4, 142.1, 149.5, 151.1, 158.8, 165.8, 166.1; IR: *ν* 3251, 1728, 1612, 1541, 1515, 1464, 1303, 1266 cm^-1^; MS (ESI): *m/z* 393.1 [M - H]^-^. 

### Synthesis of ethyl 1-benzothiazol-2-yl-4-oxo-4H-quinolizine-3-carboxylate (**11**)

2-Aminophenthiol (250 mg, 2 mmol) was added to a 50 mL of round flask charged with compound **10** (490 mg, 2 mmol) and acetic acid (15 mL). The reaction mixture was heated to reflux for about one hour till TLC confirmed that the reaction had finished. After removal of the solvent, the residue was recrystallized from ethyl acetate to offer a needle-like yellow solid. Yield：65%；m.p.: 199-200 °C; ^1^H-NMR (DMSO-*d*_6_): *δ* 1.35 (t, 3H, *J* = 7.1 Hz, CH_2_C*H*_3_), 4.32 (q, 2H, *J* = 7.1 Hz, C*H*_2_CH_3_), 7.48 (t, 1H, *J* = 7.6 Hz, Ar-*H*), 7.57 (t, 1H, *J* = 7,6 Hz, Ar-*H*), 7.70 (t, 1H, *J* = 7.2 Hz), 8.07 (d, 1H, *J* = 8.0 Hz, Ar-*H*), 8.15 (d, 1H, *J* = 8.0 Hz, Ar-*H*), 8.25 (t, 1H, *J* = 7.6 Hz, Ar-*H*), 8.75 (s, 1H, Ar-*H*), 9.47 (d, 1H, *J* = 7.2 Hz, Ar-*H*), 9.59 (d, 1H, *J* = 8.0 Hz, Ar-*H*); ^13^C-NMR (DMSO-*d*_6_): *δ* 15.2, 61.2, 105.0, 106.0, 120.0, 122.8, 123.3, 124.9, 126.1, 127.5, 131.3, 134.2, 139.4, 142.9, 144.6, 154.3, 154.7, 165.4, 165.9; IR: *v* 1736, 1670, 1623, 1583, 1496, 1222, 1133, 780 cm^-1^; MS (ESI): *m/z* 373 [M + Na]^+^.

### Synthesis of 1-benzothiazol-2-yl-4-oxo-4H-quinolizine-3-carboxylic acid (**12**)

KOH (765 mg, 13.7 mmol) dissolved in water (6 mL) was added to a 100 mL round flask charged with compound **11** (480 mg, 1.37 mmol) and ethanol (24 mL). The reaction mixture was heated to reflux for about four hours till TLC confirmed that the reaction had finished. After removal of the solvent, the residue was re-dissolved in water (30 mL) and then acidified to pH 2. The product was extracted three times with CH_2_Cl_2_ (50 mL) and the combined organic layer was dried over anhydrous MgSO_4_. After filtration of the MgSO_4_, the solvent was removed under reduced pressure and the residue was purified by recrystallization from chloroform/petroleum ether to generate **12** as a yellow solid. Yield 68%. m.p.: >300 °C; ^1^H-NMR (CDCl_3_): *δ* 7.32 (dd, 1H, *J* = 7.6 Hz, *J* = 7.2 Hz, Ar-*H*), 7.64-7.67 (m, 2H, Ar-*H*), 7.81-7.91(m, 2H, Ar-*H*), 7.95 (d, 1H, *J* = 8.0 Hz, Ar-*H*), 8.78 (d, 1H, *J* = 4.8 Hz), 9.21 (s, 1H, Ar-*H*), 9.43 (d, 1H, *J* = 7.2 Hz, Ar-*H*), 13.95 (s, 1H, COO*H*); ^13^C-NMR (DMSO-*d*_6_): *δ* 110.2, 113.2, 119.9, 120.6, 122.6, 123.3, 127.6, 128.3, 130.7, 137.3, 138.3, 138.7, 147.7, 151.4, 152.4, 163.8, 165.7; IR: *v* 1750, 1611, 1564, 1453 cm^-1^; MS (ESI): *m/z* 345.0 [M + Na]^+^, 361.0 [M + K]^+^.

### General procedure for the synthesis of compounds **13a-c**

To a 50 mL of round flask charged with phenylenediamine derivative (1 mmol) and nitrobenzene (10 mL) was added compound **10** (245 mg,1 mmol) and then the solution was slowly heated to 100 °C. After 1 h stirring, the reaction mixture was further heated to 150 °C and stirred till TLC showed the absence of starting **10**. The solution was cooled to room temperature and directly loaded onto a silica column for chromatographic purification. The eluents were first petroleum ether and then ethyl acetate/petroleum ether (1:2). After the nitrobenzene was eluted out, CHCl_3_/CH_3_OH was used to elute the corresponding **13a-c**. 

*Ethyl 1-(1H-benzimidazol-2-yl)-4-oxo-4H-quinolizine-3-carboxylate* (**13a**): Yield 85 %. m.p.: 194-195 °C; ^1^H-NMR (CDCl_3_): *δ* 1.20 (t, 3H, *J* = 7.2 Hz, OCH_2_C*H*_3_), 4.12 (q, 2H, *J* = 7.2 Hz, OC*H*_2_CH_3_), 7.21 (t, 1H, *J* = 7.2 Hz, Ar-*H*), 7.31~7.36 (m, 2H, Ar-*H*), 7.67~7.75 (m, 3H, Ar-*H*), 8.56 (s, 1H, Ar-*H*), 8.94 (d, 1H, *J* = 8.9 Hz, Ar-*H*), 9.30 (d, 1H, *J* = 7.2 Hz), 11.5 (br, 1H, N*H*); ^13^C-NMR (CDCl_3_): *δ* 14.2, 61.1, 104.8, 105.2, 117.7, 122.9, 124.8, 129.6, 135.6, 141.1, 144.6, 148.8, 155.2, 165.3; IR: *v* 1725, 1678, 1624, 1538 cm^-1^; MS (ESI): *m/z* 334 [M+1], 356 [M + Na]^+^.

*Ethyl 1-(5(6)-chloro-1H-benzimidazol-2-yl)-4-oxo-4H-quinolizine-3-carboxylate* (**13b**): Yield 83 %; m.p.: 216-217 °C; ^1^H-NMR (CDCl_3_): *δ* 1.18 (t, 3H, *J* = 7.2 Hz, OCH_2_C*H*_3_), 4.10 (q, 2H, *J* = 7.2 Hz, OC*H*_2_CH_3_), 7.23-7.29 (m, 2H, Ar-*H*), 7.63-7.76 (m, 3H, Ar-*H*), 8.57 (s, 1H, Ar-*H*), 9.04 (d, 1H, *J* = 8.8 Hz, Ar-*H*), 9.33 (d, 1H, *J* = 4.8 Hz, Ar-*H*), 11.8 (br, 1H, N*H*); ^13^C-NMR (CDCl_3_): *δ* 14.1, 61.0, 104.4, 104.9, 117.9, 123.3, 124.7, 128.3, 129.6, 135.8, 141.1, 144.5, 150.0, 155.1, 165.1; IR: *v* 3444, 3219, 1730, 1644, 1488, 1106 cm^-1^; MS (ESI): *m/z* 366 [M-1]^-^.

*Ethyl 1-(5(6)-methoxy-1H-benzimidazol-2-yl)-4-oxo-4H-quinolizine-3-carboxylate* (**13c**): Yield 88%; m.p.: 234-236 °C; ^1^H-NMR (CDCl_3_): *δ* 1.20 (t, 3H, *J* = 7.2 Hz, OCH_2_C*H*_3_), 3.89 (s, 3H, OC*H*_3_), 4.13 (q, 2H, *J* = 7.2 Hz, OC*H*_2_CH_3_), 6.95 (d, 1H, *J* = 8.8, Ar-*H*), 7.21-7.71 (m,. 3H, Ar-*H*), 8.24 (d, 1H, *J* = 8.0 Hz, Ar-*H*), 8.54 (s, 1H, Ar-*H*), 8.95 (d, 1H, *J* = 8.8 Hz, Ar-*H*), 9.30 (d, 1H, d, *J* = 7.2 Hz, Ar-*H*); ^13^C-NMR (CDCl_3_): *δ* 165.36, 156.81, 155.19, 144.55, 140.98, 135.40, 134.56, 129.53, 129.30, 124.83, 123.49, 117.66, 105.13, 105.08, 61.05, 55.85, 14.18; IR: *v* 3435, 1723, 1615, 1450 cm^-1^; MS (ESI): *m/z* 362 [M-1]^-^.

### General procedure for the synthesis of compounds **14a-c**

To a 100 mL of round flask charged with compound **13** (1 mmol) and ethanol (20 mL) was added KOH (560 mg, 10 mmol) dissolved in water (5 mL). The reaction mixtures were heated to reflux till TLC confirmed that the reactions had finished. After removal of the solvent, the residue was re-dissolved in water (20 mL) and then acidified to pH 2. The resulting suspensions were filtered, dried and purified by column chromatography (eluents: CHCl_3_/CH_3_OH) to obtain corresponding **14a-c** as yellow solids.

*1-(1H-Benzimidazol-2-yl)-4-oxo-4H-quinolizine-3-carboxylic acid* (**14a**): Yield 20 %. m.p.: >300 °C; ^1^H-NMR (DMSO-*d*_6_): *δ* 7.23 (m, 2H, Ar-*H*), 7.53 (m, 1H, Ar-*H*), 7.70 (m, 1H, Ar-*H*), 7.79 (t, 1H, *J* = 6.8 Hz, Ar-*H*), 8.28 (t, 1H, *J* = 5.6 Hz, Ar-*H*), 8.97 (s, 1H, Ar-*H*), 9.47 (d, 1H, *J* = 6.8 Hz, Ar-*H*), 9.85 (d, 1H, *J* = 9.2 Hz, Ar-*H*), 13.14 (s, 1H, N*H*), 13.75 (s, 1H, COOH); ^13^C-NMR (DMSO-*d*_6_): *δ* 104.6, 107.0, 111.8, 119.2, 120.6, 122.3, 123.3, 125.8, 130.2, 134.8, 138.1, 139.4, 143.9, 144.2, 149.1, 159.4, 166.1; IR: *v* 1705, 1612, 1497, 1443 cm^-1^; HRMS: *m/z* calcd for C_17_H_10_N_3_O_3_: 304.0718; found: 304.0727.

*1-(5(6)-Chloro-1H-benzimidazol-2-yl)-4-oxo-4H-quinolizine-3-carboxylic acid* (**14b**): Yield: 35 %. m.p.>300 °C; ^1^H-NMR (DMSO-*d*_6_) *δ*: 7.43 (dd, 1H, *J* = 8.4 Hz, *J* = 1.6 Hz, Ar-*H*), 7.74 (d, 1H, *J* = 8.4 Hz, Ar-*H*), 7.78 (s, 1H, Ar-*H*), 7.79 (t, 1H, *J* = 7.2 Hz, Ar-H) , 8.25 (t, 1H, *J* = 7.6 Hz, Ar-*H*), 8.89 (s, 1H, Ar-*H*), 9.09 (d, 1H, *J* = 8.8 Hz, Ar-*H*), 9.45 (d, 1H, *J* = 7.2 Hz, Ar-*H*); ^13^C-NMR (DMSO-*d*_6_) *δ*: 101.9, 104.9, 114.6, 116.3, 120.7, 124.9, 125.2, 129.2, 130.6, 133.9, 136.0, 138.9, 141.7, 144.2, 148.6, 158.6, 165.7; IR: *v* 3434, 1718, 1612, 1499 cm^-1^; MS (ESI) *m/z:* 338 [M-1]^-^.

*1-(5(6)-Methoxy-1H-benzimidazol-2-yl)-4-oxo-4H-quinolizine-3-carboxylic acid* (**14c**): Yield 45 %. m.p.: >300 °C; ^1^H-NMR (DMSO-*d*_6_): *δ* 3.86 (s, 3H, OC*H*_3_), 7.06 (dd, 1H, *J* = 8.4 Hz, *J* = 2.0 Hz, Ar-*H*), 7.21 (d, 1H, *J* = 2.0 Hz, Ar-*H*), 7.66 (d, 1H, *J* = 8.8 Hz, Ar-*H*), 7.80 (t, 1H, *J* = 6.8 Hz, Ar-*H*), 8.26 (t, 1H, *J* = 8.4 Hz,Ar-*H*), 8.88 (s, 1H, Ar-*H*), 9.09 (d, 1H, *J* = 8.8 Hz, Ar-*H*), 9.48 (d, 1H, *J* = 7.2 Hz, Ar-*H*); ^13^C-NMR (DMSO-*d*_6_): *δ* 165.76, 158.73, 157.63, 146.81, 144.11, 141.15, 138.64, 136.03, 130.49, 129.85, 125.03, 120.65, 115.89, 114.55, 104.84, 103.86, 97.09, 56.24; IR: *v* 3415, 1728, 1618 cm^-1^; MS (ESI): *m/z* 334 [M-1]^-^.
